# Baroreflex sensitivity in frailty syndrome

**DOI:** 10.1590/1414-431X20198079

**Published:** 2019-04-08

**Authors:** M.S.S. Buto, A.M. Catai, V. Vassimon-Barroso, M.O. Gois, A. Porta, A.C.M. Takahashi

**Affiliations:** 1 Departamento de Fisioterapia, Universidade Federal de São Carlos, São Carlos, SP, Brasil; 2 Department of Biomedical Sciences for Health, University of Milan, Milan, Italy; 3 Department of Cardiothoracic, Vascular Anesthesia and Intensive Care, IRCCS Policlinico San Donato, San Donato Milanese, Milan, Italy

**Keywords:** Baroreflex, Autonomic nervous system, Aging, Frailty

## Abstract

Frailty is related to a decrease in the physiological reserves, which causes difficulties in maintaining homeostasis. An example of physiological mechanisms for cardiovascular homeostasis is the baroreflex. The aim of this study was to compare baroreflex among frail, prefrail, and nonfrail individuals, in supine and orthostatic positions. Community-dwelling older adults were evaluated and categorized into frail, prefrail, or nonfrail groups, according to frailty phenotype. The RR interval (RRi) and systolic blood pressure (SBP) series were recorded for 15 min in the supine and 15 min in the orthostatic positions. Mean and variance of RRi and SBP, and baroreflex evaluated by phase, gain (α), and coherence (K^2^) were determined. A two-way repeated measures ANOVA, with Tukey's post hoc, was applied for group, position, and their interaction effects. The significance level established was 5%. Prefrail and frail participants did not present a significant decrease in mean values of RRi after postural challenge (893.43 to 834.20 ms and 925.99 to 857.98 ms, respectively). Frail participants showed a reduction in RRi variance in supine to orthostatic (852.04 to 232.37 ms^2^). Prefrail and frail participants showed a decrease in K^2^ after postural change (0.69 to 0.52 and 0.54 to 0.34, respectively). Frail participants exhibited lower values of K^2^ (0.34) compared to nonfrail and prefrail participants (0.61 and 0.52, respectively). Baroreflex indicated the presence of decoupling between heart period and SBP in frail and prefrail. Thus, reduced K^2^ might be a marker of the frailty process.

## Introduction

In the last few decades, researchers and health professionals have considered frailty as a distinct geriatric syndrome that shows high prevalence with increasing age (1). Frailty is described as a clinical state of vulnerability to stress, with progressive decline in the ability to maintain homeostasis (2). Although the early stages of the frailty process may be clinically silent, when decreases in physiological reserves reach a critical threshold, the organism becomes more vulnerable to stressors and risk of adverse outcomes, such as falls, hospitalization, disability, functional decline, and death, increases substantially (2).

Cardiovascular disease (CVD) has been strongly related to frailty (3,4). It seems both conditions share common pathways and each may lead to the other in a vicious cycle leading to poor outcomes over time (3). In CVD, the presence of dysregulation in the autonomic nervous system (ANS) has already been described (5), which has a crucial role in homeostasis maintenance in several physiological functions, especially the cardiovascular system (6). Thus, the difficulty in effectively adjusting the ANS to the different situations to which the individual is exposed reflects an impairment of homeostatic mechanisms. An example of a physiological mechanism for cardiovascular control is the baroreflex (7).

The major role of the arterial baroreflex is to maintain blood pressure (BP) homeostasis. BP information is sensed by stretch receptors (baroreceptors) mainly located on the wall of carotid arteries and aorta (8). In response to variations in BP, baroreceptor inputs are sent to control centers in the brainstem via afferent neural fibers, which process received inputs and modulate autonomic outflow, producing the necessary responses in cardiac contractility, vasoconstriction, and heart rate (HR) in order to guarantee the control of BP (8).

In the aging process, a decline in baroreflex sensitivity (BRS) is expected (9,10). Therefore, there is a reduction of HR responsiveness as a counterpoint to acute changes of BP and a decrease of the baroreflex capacity in buffering changes in systemic BP (8). Besides, BRS has already been used as a predictor of adverse outcomes or progression of some conditions such as coronary surgery (11), Chagas disease (12), chronic kidney disease (13), hypertension (14), myocardial infarction (15,16), and heart failure (17).

In this sense, given the relevance of this theme and the subsequent possible impact in health and economic issues (18), the characterization of prefrail and frail older adults through baroreflex assessment could contribute to the identification of risk profile to adverse outcomes, elucidate the ANS underlying mechanisms, as well as provide information for the design of specific interventions. The aim of this study was to verify if frail individuals would present an impairment in baroreflex.

## Material and Methods

### Study population and data collection

The recruitment of volunteers was carried out through posters and leaflets delivered to churches, drugstores, geriatric outpatient clinics (secondary healthcare), and primary healthcare in cities of São Paulo (Brazil). Additionally, a local database was used to invite previous research volunteers to participate in this study. Once an individual demonstrated interest, an interview was scheduled to complete anamnesis, frailty screening, and cardiovascular assessment; this was completed in the Physiotherapy Department of the Federal University of São Carlos (Brazil). Thus, the sample for this study was composed of community-dwelling older adults.

As inclusion criteria, individuals had to be 60 years of age or older, be able to comprehend the instructions, agree to participate, and present a standard electrocardiogram (ECG) without alterations at rest. Participants must not have a) cognitive impairment with scores ≤18 on the Mini-Mental State Examination (MMSE) (2), b) temporary or permanent inability to walk – use of a walker or walking cane was allowed, c) stroke, d) Parkinson's disease, e) severe hearing and vision deficits that considerably harm communication, f) be in terminal stages, g) atrial fibrillation, h) malignant ventricular arrhythmia, i) complex ectopic ventricular beat, j) sinus or supraventricular tachycardia, k) 2^o^ and 3^o^ atrioventricular block, or (l) use of a pacemaker in the resting ECG. The exclusion criteria were problems related to equipment calibration, non-stationary position, and noise in the signal.

This study was approved by the Ethics Committee of the Federal University of São Carlos (ID: 512.637/2014). Written consent was obtained from all volunteers. All procedures were performed in accordance with the ethical standards of the 1964 Helsinki Declaration.

### Anamnesis and frailty assessment

Age, anthropometric characteristics (body mass, height), years of education, medicine use, and presence of comorbidities were collected for clinical characterization. Body mass index (BMI) was calculated. Individuals were divided into three groups: nonfrail, prefrail, and frail, according to the phenotype criteria (2). These criteria were: a) low grip strength decrease: in the lowest 20% of the population at baseline, adjusted for gender and BMI; b) slow gait: in the lowest 20% of the population at baseline, based on time to walk 4.6 m, adjusted for gender and height; c) unintentional weight loss: over 4.5 kg or 5% of body weight in the prior year; d) self-reported exhaustion identified by two questions from the Center for Epidemiologic Studies – Depression Scale (CES–D): (“during the last week, did you feel you had to make an effort to cope with your usual tasks?” and “during the last week, were you not able to proceed with your duties?”); the response options “often” (about 3–4 days/week) and “always” (most of the time) in at least one question were used to indicate presence of self-reported exhaustion; e) low physical activity level: in the lowest 20% of the population, based on each volunteer's report (kcal/week), according to the Minnesota Leisure Time Activity Questionnaire, translated and adapted for use in Brazil (19).

Individuals who met three or more criteria were considered frail; one or two criteria were considered prefrail; and those who met none of the criteria were considered nonfrail.

### Procedures and experimental protocol

All volunteers were evaluated during the morning in order to minimize circadian cycle effects. The experiments were conducted in a climate-controlled (22–23°C) room with relative air humidity of 40–60%. In order to reduce volunteers' anxiety, familiarization procedures were performed so the volunteers would feel comfortable with the experimental protocols, technicians, equipment, and materials. Each volunteer was instructed to avoid caffeine and alcoholic beverages, and to avoid performing any moderate or heavy exercise on the day before participation.

The volunteers rested in the supine position for 10 min. After this period, electrocardiogram (ECG), BP, and breathing recordings were collected for 15 min. Then, the volunteers were instructed to actively change to the orthostatic position, in which they remained for 15 min. They were also instructed to breathe spontaneously, not to talk unnecessarily, and to remain awake during the test.

### Signal acquisition

The ECG signal was collected by a bioamplifier (BioAmp Power Lab, AD Instruments, Australia) with electrodes placed on the MC5 lead, and respiratory movements were captured by a respiratory belt (Marazza, Italy). The arterial BP waves were obtained by a plethysmographic arterial pressure device (Finometer PRO, Finapres Medical Systems, The Netherlands), with a cuff placed on the distal extremity of the right middle finger. The right hand was kept close to the volunteer's heart with the help of a sling, which fixed the volunteer's arm to his chest throughout the experiment. The signal acquisition frequency was sampled at 1000 Hz.

The extraction of beat-to-beat variability series was carried out according to previous descriptions (20). After extraction of the series, stable sequences of 256 points in the supine and orthostatic positions were chosen (21). Evident non-stationary series, as well as mean progressive increases or decreases, or sudden variance changes, were excluded.

### Data collection

The means and variances of the R-R interval (RRi) and BP were calculated. Baroreflex was evaluated by phase, coherence (K^2^), and gain (α). Baroreflex was calculated by cross-spectral analysis using a bivariate autoregressive model (22). The phase was computed as the phase of the cross-spectrum from BP to RRi and represents the delay between the change in BP and the subsequent change in RRi, measured in radians. The squared coherence was computed as the ratio of the squared modulus of the cross-spectrum to the product of the power spectra. Coherence was used to estimate the strength of the coupling between RRi and BP. In this study, phase and coherence were sampled at the frequency of vasomotor oscillations (Mayer waves) at the low frequency (LF) band, which oscillates between 0.04–0.15 Hz and is related to the sympathetic predominance (21,23). Gain in the LF band was calculated as the square root of the ratio of the LF power of the RRi series to that of the BP series (7) and characterizes the relationship between BP and RRi.

### Statistical analysis

The Kolmogorov-Smirnov test was used to verify the normality of the data distribution. Logarithmic transformations were then applied to all data.

In order to compare the volunteers' anthropometric characteristics and age, one-way ANOVA was applied. When a significant difference was detected, Tukey's post-hoc test was applied to identify the specific comparison. Chi-squared tests were applied to compare gender, beta blocker use, and presence of comorbidities. A two-way repeated measures ANOVA, with Tukey's post-hoc test, was applied to test the group and position effects (independent variables), and their interaction in terms of the cardiovascular variables (dependent variables). The significance level established for these tests was 5%. Statistical analysis was performed using SigmaPlot version 11.0 (Systat Software, USA).

Sample size calculation was performed *a priori* using G* Power software (version 3.1.3; Germany), which determined a sample of 21 participants (power=80%, effect size=0.4, and alpha=0.05). This calculation was performed based on two-way repeated measures ANOVA, considering effects of groups (nonfrail, prefrail, and frail), position (supine and orthostatic), and the interaction between these in terms of the cardiovascular variables.

## Results

**Figure 1:**
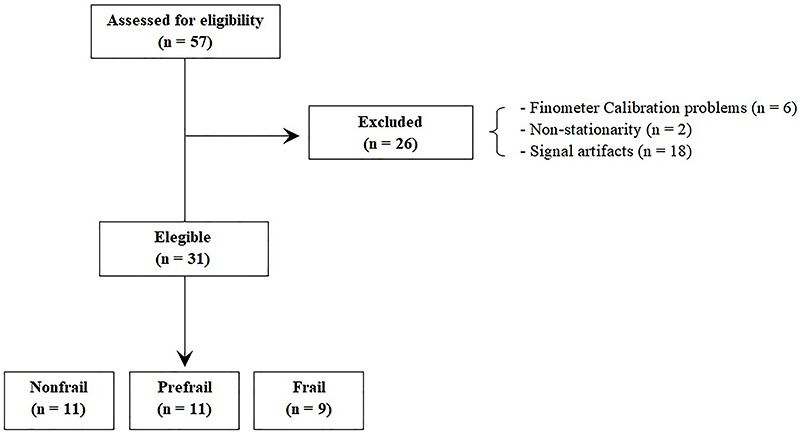
Flowchart of sample selection.

A total of 57 individuals were evaluated, 26 of which were excluded. Six participants were excluded due to problems with the Finometer calibration, two for non-stationary signals, and 18 for signal artifacts. Therefore, the final cohort was composed of 31 individuals, divided into three groups: nonfrail (n=11), prefrail (n=11), and frail (n=9) (Figure 1).

Table 1 shows the volunteers' age, and anthropometric and clinical characteristics. There was no difference in sex, age, weight, BMI, beta blocker use, or presence of comorbidities between the groups. Stature was significantly lower in the frail group compared to the prefrail group.

Table 2 shows mean and variance of RRi and BP. In terms of RRi, only the nonfrail group presented a decrease in mean values comparing supine to the orthostatic position (P<0.001). Prefrail and frail did not present an adequate response of RRi after postural challenge. In relation to variance, the frail group showed a reduction after active postural maneuver (P=0.013). None of the indices demonstrated significant difference in BP between the groups and positions.

The values related to phase, α, and K^2^ are also presented in Table 2. Regarding α, there was an effect of position (P=0.039). The prefrail and frail groups showed a significant K^2^ decrease in the orthostatic position compared to the supine position (P=0.014 and P=0.007, respectively). The frail group demonstrated significantly lower K^2^ values compared to the nonfrail (P=0.023) and prefrail groups (P=0.030). Phase did not show significant differences among groups and positions.

## Discussion

The main findings of this study were: i) the prefrail and frail groups did not reduce mean values of RRi after orthostatic challenge as expected; ii) the K^2^ values of the frail group were lower than those presented by the nonfrail and prefrail groups, which suggests an impairment in the interaction between HP and BP in the frail group; iii) prefrail and frail groups showed an antagonistic response after active postural maneuver, identified by a significant decrease in K^2^, indicating a decoupling between heart period (HP) and BP in response to postural changes.

Regarding age, and anthropometric and clinical characteristics, only stature was significantly different between prefrail and frail groups. Despite the high prevalence of hypertension and diabetes in the three groups, no significant difference was observed between them. Although a higher number of chronic diseases is related to frailty (2), our findings corroborated a previous study (24) that also observed high rates of those comorbidities in similar groups, although no difference was detected between them.

Of the cardiovascular variables, there was significant difference only in HP. The nonfrail group presented a significant decrease in mean RRi after the orthostatic challenge, which is in agreement with an expected physiological response (25,26). However, the same was not observed in the prefrail and frail groups, indicating a possible impairment in HP control.

A reduced variance of RRi is expected after postural maneuvers (26). Only the frail group presented that response. Nevertheless, a drastic drop was observed in RRi in the orthostatic position compared to the supine position. The result could reflect an adrenergic dysfunction, and in addition to the age factor, frailty would trigger an adrenergic overflow, contributing to a sympathetic over-activation (27,28) and consequently result in an exacerbated drop in variance of RRi. This inadequate response could be indicative of modifications at the morphological level, such as arterial remodeling, similar to that which occurs in CVD development; this would reinforce the strong link between frailty and CVD (28).

On the other hand, SBP values remained unaltered among the groups after postural challenge. It seems that, although the gradual loss of physiological reserve inherent to aging, which is even more aggressive in the frailty process (29,30), prefrail and frail individuals of the current study still developed a response to active postural maneuver. Thus, it is possible that the residual physiological reserve allowed individuals to develop compensatory mechanisms or had alternate pathways to achieve an effectively response (29).

It has been suggested that around a 30% loss of physiological reserve still allows good body functionality (30). When this threshold is surpassed in the frailty process and affects multiple systems, the repair mechanisms cannot maintain system homeostasis (30). Therefore, some adverse outcomes, such as orthostatic hypotension, can appear as a final consequence of the impairment of network interaction and be a sign of system dysregulation in frailty (31). These findings can be attributed to impairment of the baroreflex mechanism and not solely to BP alterations.

There is a decrease of the intensity of the causal relation between HP and BP with the aging process, leading to a situation of progressive HP-BP uncoupling with age (32) and can be featured by reduced K^2^ values. It seems that in the frailty process, this condition may overlap the aging factor and could be even more impaired. In accordance with this was the behavior presented by the frail group with lower K^2^ values compared to nonfrail and prefrail groups. Furthermore, frail and prefrail individuals presented lower K^2^ values in the orthostatic position compared to the supine position, which is an antagonistic response. In healthy individuals, an increase in K^2^ is expected during orthostatism, due to the higher gravitational effect over the baroreflex, in order to adjust to the postural change (33). Once the triggered physiological response through a postural challenge depends on integrated networks of control systems, reduced K^2^ could be a first signal of impairment in this interaction present in the frailty process.

The phase parameter seemed not to suffer changes with increasing age (34). Similarly, in the presence of frailty, this index did not change. Thus, phase was not able to differentiate the groups nor the positions.

In older adults, a decrease in baroreflex, which is evaluated by α index, is expected (10,34–36). Furthermore, some pathologies such as heart failure (37), myocardial infarction (37), coronary artery disease (34), and orthostatic hypotension (31) also present a greater reduction in this index, unlike the frailty syndrome, which does not seem to result in further reductions.

While some studies opt to evaluate BRS in the supine position, its evaluation in the orthostatic position seems to be more appropriate, as baroreceptor activation depends on BP oscillation. Besides, a provocative stressor stimulus has been recommended for frailty study because it could elicit a physiological response (38). In this way, the orthostatic position could stimulate the baroreceptors activation and provide maximal information of cardiovascular system integrity (33). Veerman et al. (35) and Laitinen (36) reported that healthy and physically active older adults have an attenuated gain response to postural change. In agreement with those studies, a position effect in gain was detected in our study. Thus, all groups seemed to present a decrease in this index in the orthostatic position and the frailty syndrome did not influence or impair the gain in response to postural change.

To date, this is the first study to evaluate the baroreflex in frailty syndrome. Coherence might be related to the frailty concept as a manifestation of impairment in interacting systems (29). Thus, the loss of information in the dynamics of systems underlies the reduction of adaptive capacity to daily stresses (29). Features of this syndrome could be detected earlier in this study if coherence analysis were used. Therefore, it is important to quantitatively identify coherence values to reinforce the pathophysiological behavior, such as in coronary heart disease (34) and heart failure (37), and to highlight the potential clinical importance of this index.

As a limitation of the study, a convenience sample was used for the recruitment of volunteers, so they are not representative of the entire population and generalization of results cannot be made. Also, the age cut-off was based on the local legislation (39).

In summary, HP values provided an indication of ANS impairment in prefrail and frail groups, once these groups did not present an expected response of HP after postural maneuver. Nevertheless, the baroreflex allowed us to more clearly detect changes in the coupling of HP and BP control systems. The prefrail and frail groups demonstrated impairment in baroreflex, identified by reduction on K^2^ values after active postural maneuver. This finding might be related to the syndrome's genesis; in other words, a loss of circulatory homeostasis that can be a reflection of an impaired interaction between regulation and control systems. Thus, reduced K^2^ might be a marker of the frailty process.

Futures studies regarding ANS integrity and the baroreflex can play an important role in following up frail individuals in clinical practice, as this syndrome may be a possible risk factor or predictor of cardiovascular adverse outcomes. In this sense, given the multidimensionality of this syndrome, it is indispensable to delineate global assessments and target specific clinical interventions for this population.

**Table 1:** Age, and anthropometric and clinical characteristics of the sample.

	Nonfrail (n = 11)	Prefrail (n =11)	Frail (n = 9)	P value
Female, n (%)	7 (63.6)	8 (72.7)	7 (77.8)	0.776
Age (years)	72.09±4.28	76.63±7.58	78.44±8.47	0.118
Weight (kg)	66.50±9.76	72.50±16.05	67.27±19.60	0.619
Stature (cm)	157.36±9.08	161.09±7.24*	151.11±7.22	0.031
BMI (kg/m^2^)	26.96±3.79	27.89±5.50	29.13±7.49	0.698
Beta blocker use, n (%)	3 (27.3)	2 (18.2)	4 (44.4)	0.431
Hypertension, n (%)	5 (45.5)	7 (63.6)	7 (77.8)	0.330
Diabetes, n (%)	2 (18.2)	4 (36.4)	3 (33.3)	0.608

Data are reported as means±SD or total of individuals (percentile). BMI: body mass index. *P<0.05 prefrail *vs* frail group (ANOVA and Tukey's *post hoc* test).

**Table 2:** Mean and variance of RRi, SBP, and baroreflex in the supine and orthostatic positions of the nonfrail, prefrail, and frail groups.

	RRi		SBP		Baroreflex		
	RRi mean (ms)	RRi variance (ms^2^)	SBP mean (mmH g)	SBP variance (mmHg)	Phase (rad)	α (ms/mmHg)	K^2^
Nonfrail							
Supine	980.39±190.19	628.11±502.46	127.91±18.46	29.57±16.93	-1.48±0.52	6.05±3.86	0.65±0.20
Orthostatic	771.42±132.15*	439.53±331.88	127.81 ±21.52	27.88±14.48	-1.27±0.68	3.21±2.25	0.61±0.13^#^
Prefrail							
Supine	893.43±131.49	577.76±433.22	136.85±18.44	29.05±19.14	-0.70±1.92	5.49±3.20	0.69±0.13
Orthostatic	834.20±151.41	592.73±465.62	138.44±28.03	48.05±23.35	-1.18±0.70	4.79±4.96	0.52±0.13*^#^
Frail							
Supine	925.99±219.68	852.04±1057.42	139.87 ±43.68	42.72±43.68	-1.45±0.92	6.07±3.31	0.54±0.25
Orthostatic	857.98±170.32	232.3 ±173.14*	134.35 ±22.60	34.67±19.31	-0.95±1.01	3.31±2.35	0.34 ±0.14*
P value							
Groups	0.911	0.955	0.284	0.435	0.396	0.853	**0.010**
Positions	**<0.001**	**0.046**	0.799	0.520	0.791	**0.039^a^**	**0.001**
Interaction	0.075	0.146	0.854	0.066	0.357	0.587	0.171

Data are reported as means±SD. RRi: RR interval; SBP: systolic blood pressure; α: gain (ms/mmHg); K^2^: coherence. *P<0.05 orthostatic *vs* supine position; ^#^P<0.05 nonfrail and prefrail *vs* frail group (Tukey's *post hoc* test). ^a^Multiple comparisons did not identify significant intragroup differences.
